# Performance of Intraoperative Assessment of Resection Margins in Oral Cancer Surgery: A Review of Literature

**DOI:** 10.3389/fonc.2021.628297

**Published:** 2021-03-30

**Authors:** Elisa M. Barroso, Yassine Aaboubout, Lisette C. van der Sar, Hetty Mast, Aniel Sewnaik, Jose A. Hardillo, Ivo ten Hove, Maria R. Nunes Soares, Lars Ottevanger, Tom C. Bakker Schut, Gerwin J. Puppels, Senada Koljenović

**Affiliations:** ^1^Department of Pathology, Erasmus MC, University Medical Center Rotterdam, Rotterdam, Netherlands; ^2^Department of Oral and Maxillofacial Surgery, Erasmus MC, University Medical Center Rotterdam, Rotterdam, Netherlands; ^3^Department of Otorhinolaryngology and Head and Neck Surgery, Erasmus MC, University Medical Center Rotterdam, Rotterdam, Netherlands; ^4^Department of Oral and Maxillofacial Surgery, Leiden UMC, Leiden University Medical Center, Leiden, Netherlands; ^5^Department of Dermatology, Erasmus MC, University Medical Center Rotterdam, Rotterdam, Netherlands

**Keywords:** oral cancer, squamous cell carcinoma, margin status, intraoperative assessment (IOA), specimen-driven, defect-driven, soft tissue, bone tissue

## Abstract

**Introduction:**

Achieving adequate resection margins during oral cancer surgery is important to improve patient prognosis. Surgeons have the delicate task of achieving an adequate resection and safeguarding satisfactory remaining function and acceptable physical appearance, while relying on visual inspection, palpation, and preoperative imaging. Intraoperative assessment of resection margins (IOARM) is a multidisciplinary effort, which can guide towards adequate resections. Different forms of IOARM are currently used, but it is unknown how accurate these methods are in predicting margin status. Therefore, this review aims to investigate: 1) the IOARM methods currently used during oral cancer surgery, 2) their performance, and 3) their clinical relevance.

**Methods:**

A literature search was performed in the following databases: Embase, Medline, Web of Science Core Collection, Cochrane Central Register of Controlled Trials, and Google Scholar (from inception to January 23, 2020). IOARM performance was assessed in terms of accuracy, sensitivity, and specificity in predicting margin status, and the reduction of inadequate margins. Clinical relevance (i.e., overall survival, local recurrence, regional recurrence, local recurrence-free survival, disease-specific survival, adjuvant therapy) was recorded if available.

**Results:**

Eighteen studies were included in the review, of which 10 for soft tissue and 8 for bone. For soft tissue, defect-driven IOARM-studies showed the average accuracy, sensitivity, and specificity of 90.9%, 47.6%, and 84.4%, and specimen-driven IOARM-studies showed, 91.5%, 68.4%, and 96.7%, respectively. For bone, specimen-driven IOARM-studies performed better than defect-driven, with an average accuracy, sensitivity, and specificity of 96.6%, 81.8%, and 98%, respectively. For both, soft tissue and bone, IOARM positively impacts patient outcome.

**Conclusion:**

IOARM improves margin-status, especially the specimen-driven IOARM has higher performance compared to defect-driven IOARM. However, this conclusion is limited by the low number of studies reporting performance results for defect-driven IOARM. The current methods suffer from inherent disadvantages, namely their subjective character and the fact that only a small part of the resection surface can be assessed in a short time span, causing sampling errors. Therefore, a solution should be sought in the field of objective techniques that can rapidly assess the whole resection surface.

## Introduction

Every year, around 350,000 new patients are diagnosed worldwide with oral cavity cancer. Oral cavity squamous cell carcinoma (OCSCC) is the most prevalent oral cavity cancer type. The worldwide mortality rate is 175,000 per year and the 5-year overall survival is 64.8% (1–4).

Surgery is the primary treatment for OCSCC. The goal of surgery is the complete resection of the tumor with an adequate resection margin (i.e., the shortest distance between the tumor border and the resection surface is > 5 mm) while preserving as much healthy tissue as possible to minimize the loss of function (such as, mastication and swallowing) and facial disfigurement. The resection margin is an important predictor for patient outcome and is the only oncological prognostic factor that pathologists and surgeons can influence (5–7).

For soft tissue, according to the Royal College of Pathologist (RCP), the resection margin is classified as clear when it is more than 5 mm, close when it is 1 to 5 mm, and positive when it is less than 1 mm (8). Clear margins are regarded as adequate, whereas close and positive margins are regarded as inadequate. For bone, the RCP indicates that a resection is adequate when the bone resection surfaces are cancer-negative (5).

It has been proven that inadequate resection margins in soft tissue result in a need for adjuvant therapy (re-excision or post-operative (chemo-) radiotherapy) (8). Adjuvant therapy brings an additional burden for the patient and results in increased morbidity and reduced quality of life (9). Furthermore, inadequate resection margins in soft tissue have a significantly negative effect (almost two fold reduction) on overall survival and disease-free survival (5, 7, 10). Patients with positive bone margins have a twofold reduction of disease-free and overall survival compared to patients with adequate bone margins (11–13).

However, achieving adequate resection margins in the oral cavity is often difficult due to its complex anatomy. During the operation the surgeon relies on pre-operative imaging, visual inspection and palpation.

Recent studies have shown that adequate margins are only achieved in a minority (15% - 26%) of the cases of soft tissue OCSCC (5, 7, 10). Segmental mandible resections have shown considerable improvement over the last years (0% - 14.6% positive bone margins). However, marginal mandible resections and partial maxillectomies still show a high rate of positive bone margins (16% - 35.7% and 44% - 60%, respectively) (11, 13–16).

These results indicate that visual inspection, palpation, and preoperative imaging do not warrant adequate tumor resection. Besides, the final margin status is only known a few days (soft tissue) or weeks (bone) after surgery. If at that point an inadequate margin is encountered, a second surgery is not an option, nor effective, because an accurate relocation of the site of an inadequate margin is almost impossible in most cases (6).

Furthermore, in the case of bone resections, an immediate bone reconstruction is performed (often with a free flap) to limit the loss of continuity and the adverse effects on function and aesthetics, making the second surgery undesirable.

Therefore, for optimal control of resection margins, the surgeon needs additional information during surgery. Intraoperative assessment of resection margins (IOARM) can provide this valuable information, enabling revision of margins (additional tissue resection) during the initial surgery to turn an inadequate resection into an adequate resection (6).

Two methods for soft tissue IOARM can be distinguished: the traditional defect-driven method and the specimen-driven method.

According to a 2005 survey, around 76% of the surgeons perform defect-driven IOARM, while only 14% perform specimen-driven IOARM during OCSCC surgery (17). However, the evidence that specimen-driven IOARM is superior to defect-driven IOARM is growing (5, 18–21). Therefore, the American Joint Committee on Cancer (AJCC) has recommended specimen-driven IOARM as the standard of care since 2017 (22).

In the traditional defect-driven approach, the surgeon samples one or more suspicious pieces of tissue from the wound bed for analysis by frozen section (FS) (i.e., a tissue sample that has been quick-frozen, cut by a microtome, and stained immediately for rapid microscopic diagnosis). The major disadvantage of defect-driven IOARM is that it can only indicate the presence of a tumor-positive margin and it cannot provide the exact margin value in millimeters. In the recently recommended specimen-driven method, the margins are assessed on the specimen by visual inspection and palpation followed by perpendicular incisions with or without sampling of tissue for FS examination (6). This approach provides immediate feedback on whether an additional resection is needed.

Here we review the performance of IOARM methods used during OCSCC surgery in predicting margin-status. The impact on patient outcome was assessed with respect to overall survival, disease-specific survival, local recurrence and the need for adjuvant therapy.

## Materials and Methods

### Search Strategy

A search was conducted in the following databases: Embase, Medline, Web of Science Core Collection, Cochrane Central Register of Controlled Trials, and Google Scholar. The following keywords and synonyms were used in the search filter: “oral cavity squamous cell carcinoma”, “resection margin” and “intraoperative”. Only studies written in English from inception of the database to the 23^rd^ of January 2020 were considered.

The studies were first assessed for eligibility based on the title and abstract. The following inclusion criteria were used: 1) the majority (> 90%) of the patients were surgically treated for OCSCC and 2) the performance of an IOARM method was investigated. The following exclusion criteria used were: 1) the study did not follow the resection margin definition of the RCP, 2) the study comprised a non-human population; 3) the study is a review, a commentary or a letter to the editor. The full text of studies that met the previous criteria was screened to extract and analyze the data.

### Data Analysis

#### Data Extraction

The included studies were divided based on the type of tissue assessed: soft tissue (group 1), and bone tissue (group 2).

The following patient and tumor characteristics were extracted independently by 3 researchers, when available: number of patients, male/female ratio (M/F), mean/median age (years), anatomical subsite, pathological TNM (pTNM) classification, and percentage of patients treated for primary disease. Type of IOARM was extracted from each of the included studies. The following IOARM performance variables were collected: true positives, true negatives, false positives, false negatives, accuracy (Acc.), sensitivity (Sens.), specificity (Spec.), positive predictive value (PPV) and negative predictive value (NPV). IOARM impact on patient outcome (e.g., overall survival (OS), disease-specific survival (DSS), local recurrence (LR) and the need for adjuvant therapy) was also collected.

#### Analysis of IOARM Performance and Impact on Patient Outcome

Based on the extracted data, IOARM sampling and interpretation errors (a), and the reduction in inadequate resections (b) were calculated.

##### Sampling and Interpretation Errors

Two types of error can occur during IOARM: sampling error (SE) and interpretation error (IE).

SE is the proportion of inadequate resections that are not identified during IOARM. It occurs due to non-representative sampling of tissue resulting in underestimation of inadequate margins (e.g., tissue is sampled from two suspicious regions but final histopathology indicates that there is a close margin in a region not regarded as suspicious during IOARM).

Interpretation error refers to incorrect diagnosis of the sampled tissue, resulting in under or overestimation of inadequate margins during IOARM.

##### Reduction of Inadequate Resections

The reduction in the number of inadequate resections (IR) based on IOARM was calculated using

$$ReductionIR(\%)=\left(\frac{IR_{i}-IRL_{Rev}}{IR_{i}}\right)\times 100$$

where:

*IR_i_* is the number of initially inadequate resections, without revision (additional resection);

*IR_Rev_* is the number of inadequate resections after revision.

## Results

**Figure 1:**
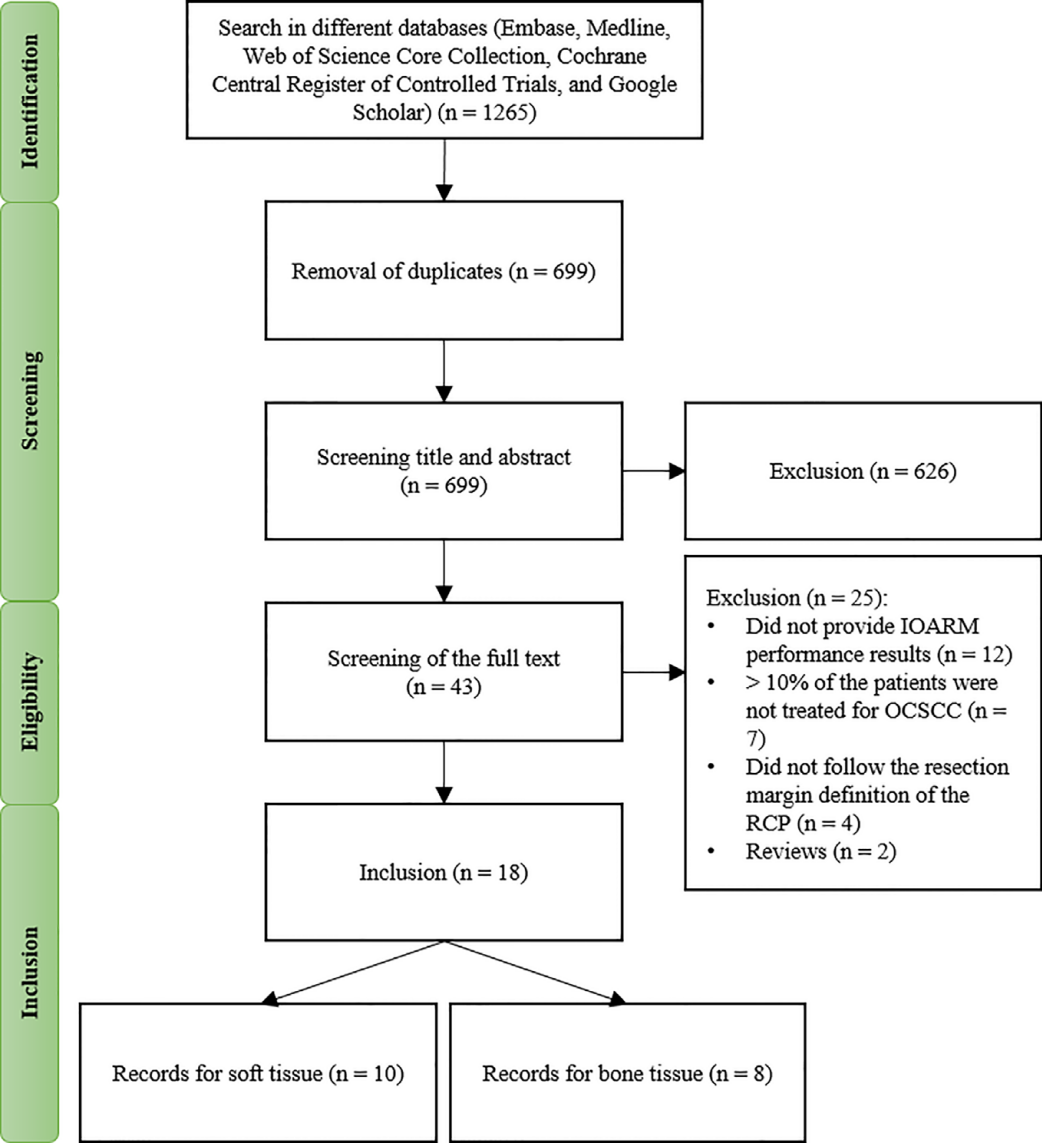
Flow diagram of the study selection process.

A total of 1265 records were found in the different databases. After removing duplicates, 699 remained and were screened on title and abstract, see **Figure 1**. This resulted in exclusion of 626 records based on the criteria applied. Of the remaining 43 records, the full text was screened resulting in further exclusion of 25 records based on the criteria of this study, as mentioned above.

### Group 1 – IOARM in Soft Tissue

Ten studies investigated the performance of IOARM methods in soft tissue (19, 23–31). The patients and tumor characteristics of the studies are shown in **Table 1**.

**Table 1:** IOARM in soft tissue: patients and tumor characteristics.

Author	Patients (N)(inclusion period)	M/F(%)	Mean age (y)	Tumor characteristics			
				Subsite(s) (%)	pT1/pT2/pT3/pT4 (%) pN0/pN1/pN2/pN3 (%)	Prior therapy(%)	Primary disease(%)
**Ord** (23)	49.0 (-)	65.0/35.0	–	Oral cavity (100.0)	30.6/16.3/14.4/38.7 -/-/-/-	0.0	–
**Pathak** (24)	416.0 (1973-2003)	58.0/42.0	64.0	Floor of the mouth (42.8); Tongue (27.6); Gingiva/Alveolus (12.0); Buccal (8.2); Retromolar trigone (1.9); Hard palate (1.2)	30.8/40.1/10.3/18.8 73.3/15.9/9.9/0.5	0.0	–
**Chaturvedi** (31)	877.0 (2007-2010)	73.0/27.0	48.0	Tongue (100.0)	18.0/45.0/18.0/19.0 65.7/19.8/14.0/0.5	0.0	100.0
**Chaturvedi** (25)	141.0 (2011-2012)	–	–	Tongue (42.2); Buccal (42.2); Lower and upper alveolus (5.7); Hard palate (2.2); Floor of the mouth (0.7); Lip (0.7); Larynx (2.8); Hypopharynx (3.5)	14.7/26.6/9.1/49.7 -/-/-/-	3.5^CT^ 14.2^S^	85.8
**Ettl** (26)	156.0 (2004-2012)	72.0/28.0	59.0	Tongue (16.0); Floor of the mouth (45.0); Cheek (7.0); Maxilla and palate (8.0); Larynx/pharynx (8.0); Alveolus (14.0)	35.3/32.0/5.8/26.9 57.0/14.1/28.9/0.0	0.0	100.0
**Buchakjian** (27)	406.0 (2005-2014)	58.0/42.0	61.0	Tongue (45.0); Lower and upper alveolus (20.0); Floor of mouth (18.0); Other (17.0)	45.0/21.0/4.0/30.0 71/10.0/19.0/<1.0	–	100.0
**Amit** (19)	51.0 (2011-2014)	61.0/39.0	59.0	Tongue (49.0); Lip (16.0); Floor of the mouth (9.0); Hard palate (5.0); Buccal (9.0); Mandible (12.0)	29.0/34.0/28.0/9.0 -/-/-/-	0.0	–
	20.0 (2011–2014)	60.0/40.0	70.0	Tongue (40.0); Lip (15.0); Floor of the mouth (10.0); Hard palate (5.0); Buccal (15.0); Mandible (15.0)	25.0/35.0/30.0/10.0 -/-/-/-		
**Mair** (28)	435.0 (2014-2015)	65.0/35.0	–	Tongue (28.5); Floor of mouth (1.0); Buccal (48.5); Lower and upper alveolus (18.2); Retromolar trigone (2.1); Lower lip (1.4)	55.9^*/^44.1^**^ 42.5^#^/57.5^##^	3.7^CT^ 3.2^RT^ 3.7^S^	96.3
**Abbas** (29)	77.0 (2010-2014)	58.0/42.0	49.0	Tongue (38.0); Cheek (37.0); Palate (8.0); Other (17.0)	–	0.0	–
**Datta** (30)	1237.0 (2012-2013)	–	–	Gingivobuccal complex (56.0); Tongue & floor of mouth (36.0); Lip (5.0); Hard palate & upper alveolus (3.0)	–	5.4^CT^ 9.7^S^	90.3

^*^Percentage of pT1 and pT2.

^**^Percentage of pT3 and pT4.

^#^Percentage of pN-.

^##^Percentage of pN+.

^CT^Percentage of patients treated with neoadjuvant chemotherapy.

^RT^Percentage of patients treated with radiation therapy prior to surgery.

^S^Percentage of patients treated with secondary surgery.

The description of the IOARM methods and their performance in the studies included are shown in **Table 2**.

**Table 2:** IOARM methods in soft tissue: description and performance.

Author	IOARM		IOARM performance									
	Method	Details of approach	Acc. (%)	Sens. (%)	Spec.(%)	PPV(%)	NPV(%)	IRi(%)	IR Rev. (%)	ReductionIR(%)	SE(%)	IE(%)
**Ord** (23)	Specimen-driven	Gross examination and FS (taken from mucosal and deep margins)	83.7	30.0	97.0	75.0	84.4	22.4	18.4	17.8	20.0	1.0
**Chaturvedi** (31)	Specimen-driven	Gross examination and FS (taken from mucosal and deep margins)	99.0	97.0	100.0	100.0	99.4	–	12.2	–	79.0	–
**Chaturvedi** (25)	Specimen-driven	Gross examination and FS (taken from mucosal and deep margins)	94.0	80.0	100.0	100.0	91.4	31.9	9.9	69.0	20.0	0.0
**Ettl** (26)	Specimen-driven	FS (taken from mucosal margins)	–	–	–	–	–	51.3	32.0	37.6	–	–
**Mair** (28)	Specimen-driven	Gross examination alone	83.7	61.9	88.3	53.1	91.2	15.6	7.4	52.6	37.0	12.8
		Gross examination and FS (taken from mucosal and deep margins)	92.9	45.5	98.8	83.3	93.5				48.0	7.7
**Datta** (30)	Specimen-driven	Gross examination and FS (taken from mucosal and deep margins)	95.4	73.1	100.0	66.0	94.8	18.8	7.8	58.5	44.3	0.0
**Pathak** (24)	Defect-driven	FS (taken from mucosal and deep margins)	–	–	70.4	–	–	–	–	–	–	–
**Buchakjian** (27)	Defect-driven	FS (taken from mucosal and deep margins)	–	48.0	72.0	57.0	65.0	37.0	18.0	51.3	64.9	10.1
**Abbas** (29)	Defect-driven	FS (taken from mucosal and deep margins)	90.9	72.7	95.3	66.6	93.9	–	–	–	27.3	5.2
**Amit** (19)	Specimen-driven	Gross examination and FS (taken from mucosal and deep margins)	–	91.0	93.0	–	–	–	16.0	–	–	–
	Defect-driven		–	22.0	100.0	–	–	–	45.0	–	–	–

The non-weighted average performance parameters for both methods were calculated over all studies that reported the necessary information (**Table 3**).

**Table 3:** The non-weighted average IOARM performance parameters for soft tissue: specimen-driven vs defect-driven method.

Performance parameters (average)	Studies using specimen-driven method* (N)	Studies using defect-drivenmethod (N)
**Accuracy (%)**	91.5 (6.0)	90.9 (1.0)
**Sensitivity (%)**	68.4 (7.0)	47.6 (3.0)
**Specificity (%)**	96.7 (7.0)	84.4 (3.0)
**^1^PPV (%)**	79.6 (6.0)	41.2 (2.0)
**^2^NPV (%)**	92.5 (6.0)	79.5 (2.0)
**^3^SE (%)**	41.4 (6.0)	46.1 (2.0)
**^4^IE (%)**	4.3 (5.0)	7.7 (2.0)

^*^Four of 6 studies were from the same institute.

^1^PPV – Positive predictive value.

^2^NPV – Negative predictive value.

^3^SE – Sampling error.

^4^IE – Interpretation error. N represents the number of studies included in the calculation.

For the specimen-driven method the reduction of inadequate resections after revision was 47.1%, based on the report of 5 studies (23, 25, 26, 28, 30). For the defect-driven method, one study has reported that the reduction in inadequate resections amounted 51.3% (27).

#### IOARM Impact on Patient Outcome

##### Overall Survival

One study reported that at 5 years follow-up there was no significant difference between defect-driven IOARM and no IOARM (p=0.836) (24). None of the other studies reported on OS.

##### Disease-Specific Survival

Pathak et al. showed that at 5 years follow-up there was no significant difference between defect-driven IOARM and no IOARM (24). None of the other studies reported on DSS.

##### Local Recurrence

Three studies reported results on LR (24, 27, 28). Two studies used defect-driven IOARM and one study used specimen-driven IOARM. For defect-driven LR of 14.4% (after 180 months of follow-up) and 23% (after 60 months of follow-up) were shown. For specimen-driven LR of 7.3% (after 14 months of follow-up) was shown (24, 27, 28). From the 3 articles reporting LR, only Pathak et al. compares the defect-driven IOARM group (supported by FS) with a control group without IOARM (22). They showed that the IOARM group had 20.1% of primary failure rate (i.e. LR), while the control group had 25.2% of primary failure rate.

##### Adjuvant Therapy

Two studies have described the influence of IOARM on the need for adjuvant therapy (19, 30). Datta et al. showed that there was no significant reduction in the need for adjuvant therapy when comparing two groups of patients, patients treated with IOARM vs patients that did not receive IOARM (30). Amit et al. reported that from all patients that underwent defect-driven IOARM 35% required adjuvant therapy. In the specimen-driven IOARM group 8% required adjuvant therapy (19).

### Group 2 – IOARM in Bone Tissue

Eight studies investigated the performance of IOARM on bone tissue (11, 12, 32–37). The patients and tumor characteristics are shown in **Table 4**.

**Table 4:** IOARM in bone tissue: patients and tumor characteristics.

Author	Patients (N) (inclusion period)	M/F(%)	Mean age (y)	Type of surgery(%)	Tumor characteristics			
					Subsite(s) (%)	pT1/pT2/pT3/pT4 (%) pN0/pN1/pN2/pN3 (%)	Prior therapy (%)	Primary disease (%)
**Forrest** (32)	16.0 (–)	–	57.0	Mandible resection: segmental (55.0); marginal (45.0)	–	–	25.0^RT^	–
**Wysluch** (33)	20.0 (2006–2007)	65.0/35.0	67.0	Segmental marginal mandibulectomy (100.0)	Floor of mouth (30.0); Retromular (50.0); Buccal (15.0); Gingiva (5.0)	–	0.0	–
**Bilodeau** (34)	27.0 (2005–2010)	63.0/37.0	59.0	Segmental mandibulectomy (100.0)	Floor of mouth (66.0); Lower and upper alveolus (19.0); Lip (4.0); Retromolar trigone (11.0)	-/-/-/100.0 37.0/19.0/44.0/-	–	85.2
**Nieberler** (35)	45.0 (2010–2013)	68.0/32.0	56.0	Segmental/ marginal mandibulectomy (88.0); partial maxillectomy (12.0)	–	–	–	–
**Namin** (36)	51.0 (2003–2013)	–	–	Mandible resection: segmental (80.0); marginal (20.0)	Oral cavity (94.0); Oropharynx (6.0)	–	18.0^RT^ 4.0^CT^	90.0
**Nieberler** (11)	102.0 (2009–2014)	69.0/31.0	62.0	Segmental/ marginal/ lingual rim mandibulectomy (86.0), partial maxillectomy (13.0); other (1.0)	Floor of mouth (41.0); Mandible (33.0); Maxilla (14.0); Cheek (7.0); Tongue (2.0); Orb. (3.0)	12.0/22.0/18.0/47.0 54.9/10.8/26.5/-	–	89.2
**Nieberler** (12)	35.0 (2012–2014)	77.0/23.0	62.0	Segmental/ partial/ lingual rim mandibulectomy (94.0); partial maxillectomy (3.0); other (3.0)	Floor of mouth (40.0); Mandible (28.6); Maxilla (5.7); Cheek (11.4); Tongue (5.7); Other (8.6)	5.7/25.7/20.0/42.9 51.4/11.4/28.6/-	–	82.9
**Cariati** (37)	17.0 (2016–2018)	71.0/29.0	69.0	Segmental mandibulectomy (100.0)	Tongue (53.0); Floor of mouth (23.5); Retromolar trigone (23.5)	-/-/-/100.0 47.0/29.0/18.0/6.0	0.0	100.0

^CT^Percentage of patients treated with neoadjuvant chemotherapy.

^RT^Percentage of patients treated with radiation therapy prior to surgery.

The description of the IOARM methods and their performance are shown in **Table 5**.

**Table 5:** IOARM methods in bone tissue: description and performance.

Author	IOARM				IOARM performance									
	Method	Sampling tool	Tissue sample	Processing technique (%)	Acc. (%)	Sens.(%)	Spec. (%)	PPV(%)	NPV(%)	IRi(%)	IR Rev(%)	Reduction IR(%)	SE(%)	IE(%)
**Forrest** (32)	Specimen-driven	Currette	bone marrow	FS (100.0)	93.8	66.7	100.0	100.0	92.9	18.8	6.3	66.5	0.0	6.3
**Wysluch** (33)	Specimen-driven	Trephine drill technique	cortical bone	FS (100.0)	–	77.0	90.0	–	–	–	–	–	–	–
**Nieberler** (35)	Specimen-driven	Cytobrush	bone marrow	FS (100.0)	96.0	80.0	98.0	80.0	97.0	11.0	2.2.0	80.0	20.0	2.2
**Namin** (36)	Specimen-driven	Currette	bone marrow	FS (100.0)	100.0	100.0	100.0	100.0	100.0	19.0	0.0	100.0	0.0	0.0
**Nieberler** (11)	Specimen-driven	Cytobrush	bone marrow	FS (100.0)	99.0	88.9	100.0	100.0	98.9	8.8	2.9	67.1	11.0	0.0
**Nieberler** (12)	Specimen-driven	Cytobrush	bone marrow	Fixation with cold methanol (59.0); Papanicolau staining (41.0)	94.0	78.0	100.0	100.0	92.9	–	–	–	22.2	0.0
**Bilodeau** (34)	Defect-driven	Currette	bone marrow; Inf. alveolar nerve	FS (100.0)	89.0	50.0	100.0	100.0	87.5	–	–	–	50.0	3.7
**Cariati** (37)	Defect-driven	Currette	bone marrow	FS (100.0)	76.5	33.3	85.7	33.3	85.7	17.6	11.8	33.0	66.7	11.8

The non-weighted average performance parameters for both methods were calculated over all studies that reported the necessary information (**Table 6**).

**Table 6:** The non-weighted average IOARM performance parameters for bone tissue: specimen-driven vs defect-driven method.

Performance variables (average)	Studies using specimen-driven method (N)	Studies usingdefect-driven method (N)
**Accuracy (%)**	96.6 (5.0)	82.8 (2.0)
**Sensitivity (%)**	81.8 (6.0)	41.7 (2.0)
**Specificity (%)**	98 (6.0)	92.9 (2.0)
**^1^PPV (%)**	96 (5.0)	66.7 (2.0)
**^2^NPV (%)**	96.3 (5.0)	86.6 (2.0)
**^3^SE (%)**	10.6 (5.0)	58.5 (2.0)
**^4^IE (%)**	1.7 (5)	7.8 (2)

^1^PPV – Positive predictive value.

^2^NPV – Negative predictive value.

^3^SE – Sampling error.

^4^IE – Interpretation error. N represents the number of studies included in the calculation.

For the specimen-driven method the reduction of inadequate resections after revision was 78.4%, based on the report of 4 studies (11, 32, 35, 36). For the defect-driven method, one study has reported that the reduction in inadequate resections amounted 33% (37).

#### IOARM Impact on Patient Outcome

##### Overall Survival

Nieberler et al. demonstrated that at 3 years follow-up OS was higher for patients treated with specimen-driven IOARM compared to the control group (OS: 70% vs 20%, respectively) (11). None of the other studies reported on OS.

##### Disease-Specific Survival

Nieberler et al. showed that at 3 years follow-up disease-free survival was higher for patients treated with specimen-driven IOARM compared to the control group (DSS: 80% vs 40%, respectively) (11). None of the other studies reported on DSS.

##### Local Recurrence

None of the studies demonstrated the impact of IOARM on LR.

##### Adjuvant Therapy

Nieberler et al. have also demonstrated that the group of patients treated with specimen-driven IOARM had a slightly lower rate of adjuvant therapy than the control group (52% RT vs 58% RT, respectively) (11). None of the other studies reported on the impact of IOARM on adjuvant therapy.

## Discussion

Surgical treatment of OCSCC patients aims for complete tumor resection with adequate margins, which is the most important prognostic factor. This goal is seldom achieved, underlining that insufficient intraoperative information is available for optimal control of resection margins. IOARM can provide such information.

Here we review the literature reporting on IOARM in OCSCC surgery. The performance of different IOARM methods in predicting margin-status, and their impact on patient outcome were studied.

Despite the pressing need for improving OCSCC surgery, only 18 studies were found that have reported on the performance of IOARM methods; 10 regarding soft tissue resection margins, and 8 regarding bone resection margins.

Of the 10 studies that investigated the performance of IOARM for soft tissue, 6 reported on the specimen-driven method, 3 on the defect-driven method and one on both.

In the majority of the specimen-driven studies (4/6), the assessment was performed by gross examination of mucosal and deep margins, followed by FS analysis of locations judged suspicious for inadequate margins (25, 28, 30, 31).

Mair et al. have assessed whether gross examination alone can be as accurate as gross examination combined with FS analysis and found no statistically significant difference in overall incidence of inadequate margins in both groups (28).

In the 3 defect-driven IOARM-studies inspection of the wound bed by the surgeon followed by FS analysis of suspicious mucosal and deep margins was performed (19, 27, 29).

Patient outcome parameters are negatively affected by inadequate resections (5, 7, 10). The studies show that IOARM improves the rate of adequate operations and as a result leads to a decrease in adjuvant therapy. Amit et al. explicitly excluded patients that received adjuvant therapy for other reasons than inadequate resections and showed that of all patients that underwent defect-driven IOARM, 35% required adjuvant therapy while only 8% of all patients that underwent specimen-driven IOARM required adjuvant therapy (19). Only Datta et al. has compared results of adjuvant therapy between patients who received IOARM and those who did not (i.e., control group). The authors demonstrated there was no significant reduction. This result can be explained by the fact that some patients receive adjuvant therapy for other reasons than an inadequate resection (e.g., extra-capsular spread and perineural involvement) (30). Future studies should be designed to study the impact of IOARM by also including the need for adjuvant therapy, next to other prognostic parameters (e.g., LR, RR, OS, DSS).

Of the 8 studies that investigated the performance of IOARM for bone tissue, 6 reported on the specimen-driven method and 2 on the defect-driven method. Cytological methods were developed for this.

Nieberler et al. demonstrated that the 3 years disease-free survival and overall survival were higher for patients treated with specimen-driven IOARM compared to the control group (DSS: 80% vs 40%; OS: 70% vs 20%). They have also demonstrated that based on specimen-driven IOARM of bone resection margins a number patients did not need to receive adjuvant radiotherapy (11).

When comparing specimen-driven IOARM with defect-driven IOARM we can conclude that for both, soft tissue and bone tissue, the SE and IE are higher for defect-driven IOARM, **Tables 3** and **6**. Consequently, the performance (e.g., average accuracy, sensitivity, specificity, PPV and NPV) of specimen-driven IOARM is better (**Tables 3** and **6**). However, it is important to stress that this conclusion is limited by the low number of available studies reporting performance results for defect-driven IOARM.

Another interesting finding was the discrepancy in the reported rate of initially adequate resections for soft tissue specimens. Some recent studies, report adequate resections in only a small minority (15%-26%) of the cases (5, 7, 10). Other studies have shown much higher rates of adequate resections, varying from 48.7% to 81.2% (23, 25–28, 30, 31).

Differences in oral subsite of the tumor might be a reason for this discrepancy. While in Asian countries, a large proportion of the patients have buccal SCC, in Europe and North-America, patients are more often treated for tongue SCC. It has been shown that tongue SCC is significantly more aggressive (more often poorly differentiated) compared to buccal SCC (38). It is harder to achieve a complete resection in poorly differentiated SCC (39). Moreover, differences in surgical approach may play a role; i.e. a difference in balancing the need to remove the tumor, while sparing healthy tissue. However, this information is not available in the papers that were studied.

This literature review shows that there is a low number of studies on the performance of IOARM available. This is the main limitation of this study. However, we firmly believe that with upcoming awareness on the need for IOARM there will be enough evidence in the literature to perform a thorough systematic review/meta-analysis, in the near future. Another limitation of this review is that the studies included performed IOARM according to different protocols. Moreover, the outcome was often evaluated according to different criteria. This makes a comparison of the studies unreliable.

Nevertheless, some conclusions can be drawn: IOARM improves patient outcome and the performance of specimen-driven IOARM is superior to the performance of defect-driven IOARM.

There can be no doubt that IOARM reduces the rate of inadequate margins (average IR Rev. for soft tissue: 47.8%; average IR Rev. for bone tissue: 78.4%), but it still shows low sensitivity (average Sens. for soft tissue: 62.1%; average Sens. for bone tissue: 71.7%) caused by a high SE (average SE for soft tissue: 42.6%; average SE for bone tissue: 24.3%), **Tables 3** and **6**. The best-performing method; specimen-driven IOARM, is logistically demanding and time-consuming. In addition, grossing fresh tissue is counter-intuitive to most pathologists for fear of interfering with final pathologic assessment. This will continue to stand in the way of IOARM widespread adoption, despite the significant improvement in OCSCC resection results, unless standard protocols and educational programs exist. At our institute we have a comprehensive IOARM protocol including a relocation protocol (6, 40).

The development of objective technology is needed to address these practical hurdles and key to facilitating specimen-driven IOARM in OCSCC. An example of such technology is Raman spectroscopy; an optical technique which has been shown to discriminate between OCSCC and surrounding healthy tissue with high sensitivity and specificity (soft and bone tissue) (41–43). A dedicated instrument employing a fiber optic needle probe for rapid assessment of resection margins on OCSCC specimen is currently under development (44).

## Author Contributions

EB, YA, and LS designed the study, extracted data, carried out the data analysis and drafted the manuscript. IH, HM, AS, JH, MN, and LO revised the manuscript critically for important intellectual content. TS, GP, and SK designed the study, supervised the research group and revised the manuscript critically for important intellectual content and gave final approval of the version to be published. All authors contributed to the article and approved the submitted version.

## Funding

We want to thank Dutch Cancer Society (Project 106467 - Optimizing surgical results for oral squamous cell carcinoma by intra-operative assessment of resection margins using Raman spectroscopy) and Eurostars E! (Project 12076 – RA-SURE) for the financial support.

## Conflict of Interest

The authors declare that the research was conducted in the absence of any commercial or financial relationships that could be construed as a potential conflict of interest.

## Abbreviations

IOARM, Intraoperative assessment of resection margins; OCSCC, Oral cavity squamous cell carcinoma; SCC, Squamous cell carcinoma; FS, Frozen sections; RCP, Royal College of Pathologists; AJCC, American Joint Committee on Cancer; M, Male; F, Female; TNM, Tumor, lymph nodes, metastasis (according to the TNM Classification of Malignant Tumors); Acc., Accuracy; Sens.. Sensitivity; Spec., Specificity; PPV, Positive predictive value; NPV, Negative predictive value; OS, Overall Survival; DSS, Disease-specific survival; LR, Local recurrence; RT, Radiotherapy.
